# Universal Pharmacare and Contraceptive Dispensations Among Youth

**DOI:** 10.1001/jamapediatrics.2025.2585

**Published:** 2025-08-18

**Authors:** Amanda K. Downey, Steven E. Hanna, Mitchell A. Levine, Laura Schummers, G. Emmanuel Guindon

**Affiliations:** 1 Department of Health Research Methods, Evidence and Impact, McMaster University, Hamilton, Ontario, Canada; 2 St Joseph’s Healthcare Hamilton, McMaster University, Hamilton, Ontario, Canada; 3 Centre for Health Economics and Policy Analysis, McMaster University, Hamilton, Ontario, Canada; 4 Collaboration for Outcomes Research and Evaluation, Faculty of Pharmaceutical Sciences, University of British Columbia, Vancouver, British Columbia, Canada; 5 Department of Economics, McMaster University, Hamilton, Ontario, Canada

## Abstract

**Importance:**

Previous studies have suggested that removing financial barriers to contraception could help reduce unintended pregnancy.

**Objective:**

To assess whether introduction of universal public funding for prescription contraception in Ontario (OHIP+) for individuals younger than 25 years and the amended program, which limited public funding to those without private insurance (OHIP−), is associated with changes in contraceptive dispensations.

**Design, Setting, and Participants:**

Interrupted time-series analyses were used to evaluate whether implementation of either policy was associated with changes in monthly contraceptives dispensed. The setting included a national database on contraceptives dispensed from retail pharmacies between September 2016 and February 2020; data analysis was performed from May 2022 to 2024. Participants included Ontario females aged 15 to 24 years to whom prescriptions were dispensed (intervention) vs controls: (1) Canadian females aged 15 to 24 years, excluding Ontario, and (2) Ontario females aged 25 to 49 years.

**Exposures:**

Implementation of free prescription contraception through OHIP+ (January 2018-March 2019) and OHIP− (April 2019-February 2020).

**Main Outcomes and Measures:**

Monthly dispensations of intrauterine devices (IUDs) and oral contraceptive pills (OCPs) per 1000 females overall and by area-level socioeconomic status (SES).

**Results:**

After OHIP+, there was an immediate level increase in IUDs dispensed to Ontario females aged 15 to 24 years (intervention) of 0.50 (95% CI, 0.15-0.84) vs 0.03 (95% CI, −0.26 to 0.32) in Canadian females aged 15 to 24 years—a relative increase of 0.48 (95% CI, 0.02-0.91). There was an immediate level increase in OCPs dispensed to Ontario females aged 15 to 24 years of 22.3 (95% CI, 14.8-29.8) vs 7.57 (95% CI, 3.07-12.1) in those aged 25 to 49 years—a relative increase of 14.8 (95% CI, 6.15-23.4). There were no statistically significant changes in monthly dispensation trends after OHIP+ and no statistically significant changes after OHIP−. In areas with lower SES, there was a significant increase in the level for IUDs of 0.64 (95% CI, 0.02-1.26) and for OCPs of 13.2 (95% CI, 1.33-25.0) after OHIP+, and a significant decrease in the level for IUDs of 0.82 (95% CI, −1.55 to −0.09) after OHIP− in Ontario vs Canadian females aged 15 to 24 years. No statistically significant changes in IUD or OCP dispensations were observed in areas with higher SES vs controls.

**Conclusions and Relevance:**

Results reveal that providing comprehensive and confidential access to prescription contraceptives was associated with increased dispensations among Ontario youth.

## Key Points

**Question:**

Was the implementation of a publicly funded pharmacare program for individuals younger than 25 years in Ontario associated with an increase in prescription contraceptive dispensations?

**Findings:**

In this time-series analysis of contraceptives dispensed from Canadian pharmacies, implementation of universal pharmacare was associated with increases in contraceptives dispensed, including intrauterine devices and oral contraceptives. The magnitude of increase was greater in areas with low socioeconomic status.

**Meaning:**

Results suggest that the implementation of universal funding for contraception may increase the use of prescription contraceptives among youth, a group at high risk of unintended pregnancy.

## Teaser

This time-series analysis study evaluates the association between a publicly funded pharmacare program for individuals younger than 25 years and an increase in prescription contraceptive dispensations.

## Introduction

Previous literature has shown that having prescription drug insurance and lower drug cost sharing were associated with increased drug use and lower health care service utilization.^1,2^ The impact of drug insurance and cost sharing has also been reported in pediatric populations where provision of insurance to previously uninsured children was associated with increased use of prescription drugs and primary care.^3,4,5^ Furthermore, cost reductions and provision of contraceptive subsidies have been associated with increased contraceptive use and decreased unintended pregnancies in adolescent and young adult populations globally.^6,7,8,9^ Previous studies have also shown improved health outcomes for previously uninsured youth enrolled in a publicly funded drug plan.^10,11^

In Ontario, drug insurance, low drug cost sharing, and adequate family income were associated with increased prescription drug use in youth,^3^ and in Manitoba, increased cost sharing was associated with an inverse effect on asthma medications dispensed in youth.^12^ The Canadian Pediatric Society (CPS) has since released a position statement calling for universal drug coverage for individuals younger than 25 years.^13^ In addition, recognizing the financial barriers and potential confidentiality breaches that young females may face when obtaining contraception, the CPS also recommended that contraception be made available confidentially at no cost until the age of 25 years.^14^ These statements echo long-standing calls by the American College of Obstetricians and Gynecologists.^15^

In January 2018, Ontario introduced OHIP+, a universal pharmacare program for all prescription drugs listed on the public drug formulary for youth younger than the age of 25 years without cost sharing, including individuals already covered via private drug plans. This program was revised in April 2019 to cover only those who did not have access to private insurance (OHIP−), reintroducing cost sharing for privately covered individuals.^16^ As a result, many Ontario youth reverted to their parent’s private drug plan,^17,18,19^ potentially compromising confidential access to prescription medication, including contraceptives.

Since its implementation, a small number of studies have investigated the impact of OHIP+ and OHIP− on drug utilization and health outcomes in youth. Initial results suggest OHIP+ may be associated with an immediate increase in the level of prescription drugs dispensed and that these effects may be more pronounced in individuals with low socioeconomic status (SES) and those aged 20 to 24 years. Reported changes to the overall trend of prescriptions dispensed in Ontario youth have been minimal after the implementation of OHIP−.^20,21,22,23^

Prescription contraceptives are frequently dispensed to individuals younger than the age of 25 years.^24^ Hormonal intrauterine devices (IUDs), a form of long-acting reversible contraception, are among the most effective contraceptives^9,25^; however, they also have the highest up-front cost.^9,26^ Financial barriers have been cited as one of the reasons young Canadian females chose less effective methods of contraception, placing them at increased risk for an unintended pregnancy.^9,14,27,28^ The possible financial incentives and impacts to confidentiality resulting from OHIP*+* and OHIP− on contraceptive use are currently unknown. The main objective of this study was to examine the association between of OHIP+ and OHIP− and contraceptives dispensed in Ontario females aged 15 to 24 years by evaluating changes in prescription contraceptives dispensed after the introduction of OHIP+, revision to OHIP−, and variability in changes by area-level SES.

## Methods

### Dataset

Research ethics board approval was not required for this cohort study. The Tri-Council Policy Statement states that “REB review is not required for research involving only anonymous data sets.”^29^ We used IQVIA’s Geographic Prescription Monitor (GPM) database to conduct this analysis. The GPM database has established validity^30^ and has been used in previous health services research.^31,32,33,34,35^ Using a panel of approximately 6100 Canadian retail pharmacies, IQVIA captures more than 75% of total prescriptions dispensed nationally. Captured prescriptions are projected to be representative of national levels; data are extrapolated to represent the entire Canadian population. Monthly estimates are created using IQVIA’s proprietary geospatial projection methods for all dispensations.^31,32,35^ The database provides monthly panel data of counts of each prescription product dispensed by product, sex, age, area-level SES (median total income of households, before tax, from the 2016 Census, in quintiles, measured at the Forward Sortation Area of the pharmacy), and payer type. In most cases, the sampling error of this dataset does not exceed 10%.^36^ The unit of analysis was prescriptions dispensed per month at retail pharmacies.

The study population included females seeking prescriptions indicated for contraception. We excluded prescriptions dispensed to males and to females younger than 15 years or older than 49 years. We excluded oral contraceptives with no estrogen due to the low number of prescriptions dispensed to younger individuals as well as contraceptive patches, rings, creams, foams, gels, and emergency contraception as these methods are not covered by OHIP+. The contraceptive implant was not available in Ontario during the time frame of this analysis. Copper IUDs are considered medical devices and are not eligible for OHIP+; therefore, copper IUDs were excluded from this study. We conducted primary analyses on hormonal IUDs and combined (estrogen/progestin) oral contraceptive pills (OCPs). This study followed the Strengthening the Reporting of Observational Studies in Epidemiology (STROBE) reporting guidelines.

### Exposure and Outcomes

The primary exposure was the policy era (ie, OHIP+ or OHIP−). Outcomes were monthly contraceptives dispensed. We calculated the rate of dispensations per 1000 females for each contraceptive type per month within age categories for each province. All rates were standardized using annual population estimates from Statistics Canada. Because population estimates are reported by year, we linearly interpolated monthly population estimates. Our primary outcome of interest was the contraceptive dispensation rates of IUDs and OCPs in Ontario females aged 15 to 24 years.

### Statistical Analysis

First, we used interrupted time-series (ITS) analyses to evaluate changes in dispensations associated with OHIP+ and OHIP−. This methodology uses a quasi-experimental design to evaluate the effects of an intervention introduced at a specific point of time^37,38^ and has been used to examine the effect of drug insurance expansion and changes in drug cost sharing in Canada, including the expansion of drug insurance in Québec,^39,40^ changes in drug cost sharing in Nova Scotia^41^ and Québec,^42,43^ and the introduction of copayments in 2002 and of income-based deductible and coinsurance in 2003 in British Columbia.^44,45,46,47,48,49^ In this analysis, the preintervention trend projected into the treatment period serves as the counterfactual, with the intervention serving as its own control.^37,38^

Second, we used controlled ITS (CITS) analyses. CITS overcomes bias that could result from cointerventions by deriving the counterfactual using the preintervention trend in the intervention group and also control group(s) expected to experience cointerventions but not the intervention.^50,51,52,53^ We used 2 control groups: (1) females aged 15 to 24 years across Canada and (2) Ontario females aged 25 to 49 years.

We stratified all analyses by age group within the intervention group (15- to 19-year-olds and 20- to 24-year-olds separately) and by SES quintile. For SES-stratified analyses, we calculated dispensation rates by dividing population estimates by 5.

For all analyses, segmented regression models were fit using an ordinary least squares model with Newey-West SEs and adjusted for serial autocorrelation. CITS models followed the same form, with additional interaction terms for the difference between the treatment and the control in the level and slope of the outcome variable after the intervention.^50^ Autocorrelation was assessed using the Cumby-Huizinga test for autocorrelation; *P* values < .05 indicated autocorrelation.^50,54^

We conducted sensitivity analysis by removing the month immediately before the policy change and the month of the policy change to control for anticipatory changes in contraceptive dispensations. We used the month after the policy changes (ie, February 2018 and May 2019) as pseudointervention dates. All analyses were conducted from May 2022 to 2024 using Stata, version 17.0 (StataCorp).

## Results

A total of 44 910 929 OCPs and IUDs were dispensed in Canada between September 2016 and February 2020. Key descriptive statistics are presented in the Table.

**Table.** Characteristics of Prescription Contraceptives Dispensed According to Policy Period

Characteristic	Pre-OHIP+, September 2016-December 2017	OHIP+, January 2018-March 2019	OHIP−, April 2019-February 2020
**Ontario females aged 15-24 y**			
No.	938 805	1 210 618	861 740
Prescription contraceptives (average per mo)			
IUDs	17 180 (1074)	30 891 (2059)	23 167 (2106)
OCPs	921 625 (57 602)	1 179 727 (78 648)	838 573 (76 234)
Socioeconomic status, IUDs			
Lowest quintile	4804	8707	5957
Low-middle quintile	4125	7488	5339
Middle quintile	2880	4881	3896
High-middle quintile	2841	5302	4388
Highest quintile	2530	4513	3587
Socioeconomic status, OCPs			
Lowest quintile	201 029	263 216	182 242
Low-middle quintile	193 029	250 913	177 164
Middle quintile	167 379	211 319	150 921
High-middle quintile	178 550	227 321	162 461
Highest quintile	181 638	226 958	165 785
**Canadian females aged 15-24 y** ^a^			
No.	2 901 313	2 935 567	2 099 864
Prescription contraceptives (average per mo)			
IUDs	41 538 (2596)	46 085 (3072)	36 183 (3289)
OCPs	2 859 775 (178 736)	2 889 482 (192 632)	2 063 681 (187 607)
Socioeconomic status, IUDs			
Lowest quintile	9893	11 115	8697
Low-middle quintile	8989	9686	7381
Middle quintile	8650	9301	7541
High-middle quintile	8396	9538	7266
Highest quintile	5610	6445	5298
Socioeconomic status, OCPs			
Lowest quintile	589 276	586 235	416 701
Low-middle quintile	597 947	607 748	429 447
Middle quintile	600 290	613 108	435 875
High-middle quintile	603 846	606 172	436 766
Highest quintile	468 416	476 219	344 892
**Ontario females aged 25-49 y**			
No.	1 391 302	1 471 578	1 039 128
Prescription contraceptive (average per mo)			
IUDs	59 579 (3724)	69 781 (4652)	53 519 (4865)
OCPs	1 331 723 (83 233)	1 401 797 (93 453)	985 609 (89 600)
Socioeconomic status, IUDs			
Lowest quintile	13 305	16 207	12 151
Low-middle quintile	13 756	16 167	12 013
Middle quintile	10 981	12 816	10 039
High-middle quintile	10 591	12 504	10 020
Highest quintile	10 946	12 087	9296
Socioeconomic status, OCPs			
Lowest quintile	286 787	306 715	216 474
Low-middle quintile	281 037	298 572	208 507
Middle quintile	270 475	280 767	199 944
High-middle quintile	249 002	264 170	185 235
Highest quintile	244 422	251 573	175 449

Abbreviations: IUD, intrauterine device; OCP, oral contraceptive pill.

^a^ Refers to prescriptions dispensed in females from all Canadian provinces, excluding Ontario.

### OHIP+/− and IUD Dispensations in Ontario Females Aged 15 to 24 Years

**Figure 1.**
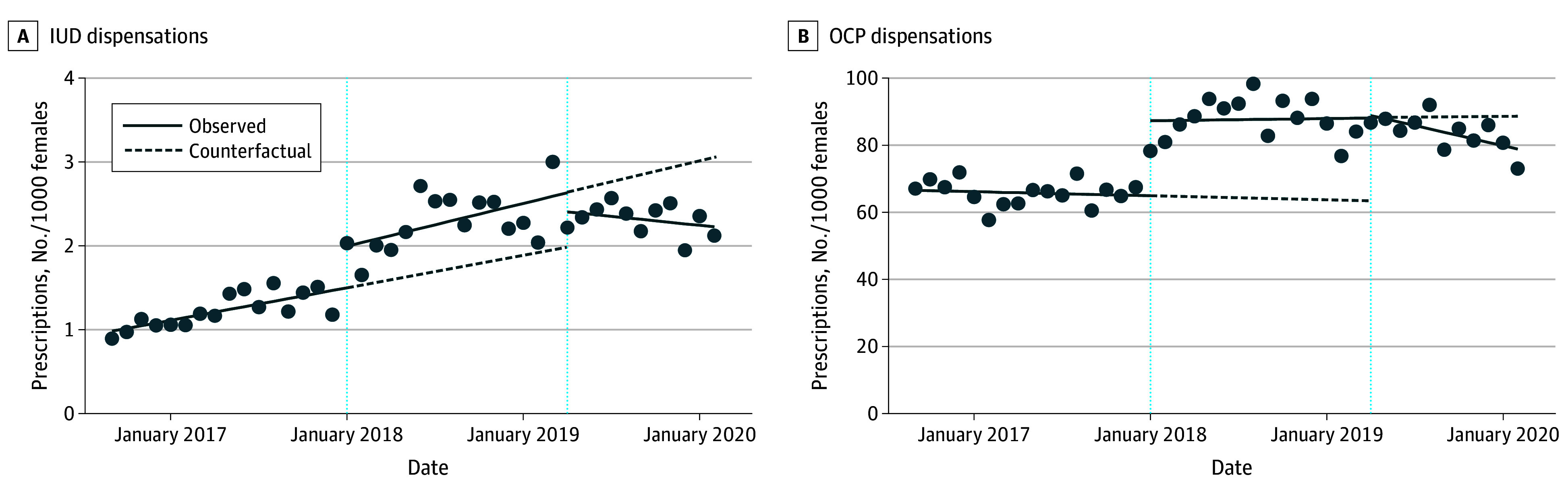
Single Interrupted Time-Series Analysis of Intrauterine Device (IUD) and Oral Contraceptive Pill (OCP) Dispensation Rate in Ontario Females Aged 15 to 24 Years

Before the introduction of OHIP+, IUD dispensations were increasing at a monthly rate of 0.03 per 1000 females (95% CI, 0.02-0.05). At the introduction of OHIP+, the level of dispensations increased by 0.50 per 1000 females (95% CI, 0.15-0.84) vs 0.03 (95% CI, −0.26 to 0.32) in Canadian females aged 15 to 24 years—a relative increase of 0.48 (95% CI, 0.02-0.91). The level of dispensations continued to increase monthly by 0.04 per 1000 females (95% CI, 0-0.08), a monthly increase of 0.01 (95% CI, −0.03 to 0.05) relative to the prepolicy era. At the introduction of OHIP−, estimated use decreased immediately by 0.23 per 1000 females (95% CI, −0.67 to 0.21) and continued to decrease monthly by 0.02 per 1000 females (95% CI, −0.05 to 0.02), a monthly decrease of 0.06 (95% CI, −0.11 to −0.01) relative to the OHIP+ period (Figure 1A). Quantitative results of the single-group ITS analyses are presented in eTable 1 in Supplement 1.

**Figure 2.**
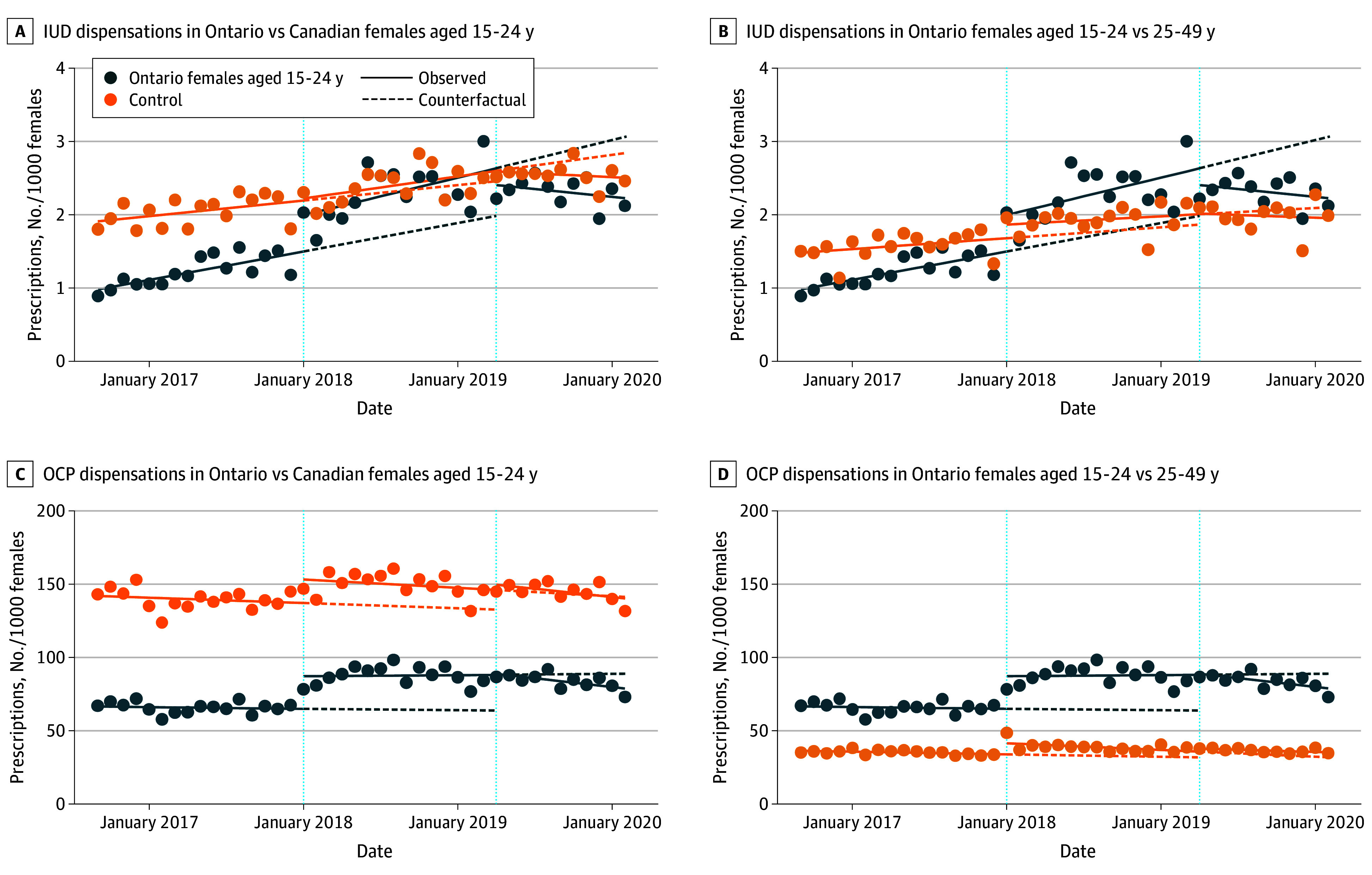
Controlled Interrupted Time-Series Analysis of Intrauterine Device (IUD) and Oral Contraceptive Pill (OCP) Dispensation Rate in Ontario Females Aged 15 to 24 Years

Our CITS results showed an increase in the level of IUD dispensations of 0.47 per 1000 (95% CI, 0.02-0.91) after OHIP+ in Ontario vs Canadian females aged 15 to 24 years. There was no statistically significant change in the monthly rate of IUD dispensations between the 2 groups after OHIP+. After OHIP−, there was a decrease in the level of IUD dispensations of 0.22 per 1000 (95% CI, −0.72 to 0.28) and the monthly dispensation rate of 0.03 per 1000 (95% CI, −0.09 to 0.03) vs the OHIP+ period in Ontario vs Canadian females aged 15 to 24 years (Figure 2A and B). All quantitative results of the CITS are presented in eTable 2 in Supplement 1.

### OHIP+/− and OCP Dispensations in Ontario Females Aged 15 to 24 Years

Oral contraceptive dispensations were decreasing at a rate of 0.10 per 1000 per month (95% CI, −0.45 to 0.25) before OHIP+. At the introduction of OHIP+, the level of dispensations increased by 22.3 per 1000 (95% CI, 14.8-29.8) vs 7.57 (95% CI, 3.07-12.1) in those aged 25 to 49 years—a relative increase of 14.8 (95% CI, 6.15-23.4). The level of dispensations continued to increase monthly by 0.06 per 1000 (95% CI, −0.78 to 1.08), a monthly increase of 0.16 (95% CI, −0.78 to 1.08) relative to the prepolicy era. At the introduction of OHIP−, there was an increase in the level of dispensations of 0.74 per 1000 (95% CI, −7.84 to 9.32), followed by a decrease in monthly dispensations by 1.00 prescription per 1000 (95% CI, −1.73 to −0.27), a monthly decrease of 1.05 (95% CI, −2.18 to 0.07) relative to the OHIP+ period (Figure 1B).

Our CITS models found an immediate increase in the level and monthly rate of OCP dispensations after OHIP+ in Ontario women aged 15 to 24 years vs those aged 25 to 49 years of 14.8 (95% CI, 6.15-23.4) and 0.38 (95% CI, −0.65 to 1.42) per 1000, respectively. The introduction of OHIP− was associated with a decrease in the level and monthly rate of OCP dispensations of 1.23 (95% CI, −10.3 to 7.87) and 1.19 (95% CI, −2.42 to 0.04) per 1000 in Ontario women aged 15 to 24 years vs those aged 25 to 49 years compared with the OHIP+ period (Figure 2C and D).

### OHIP+/− and Contraceptive Dispensations in Ontario Females Aged 15 to 24 Years

**Figure 3.**
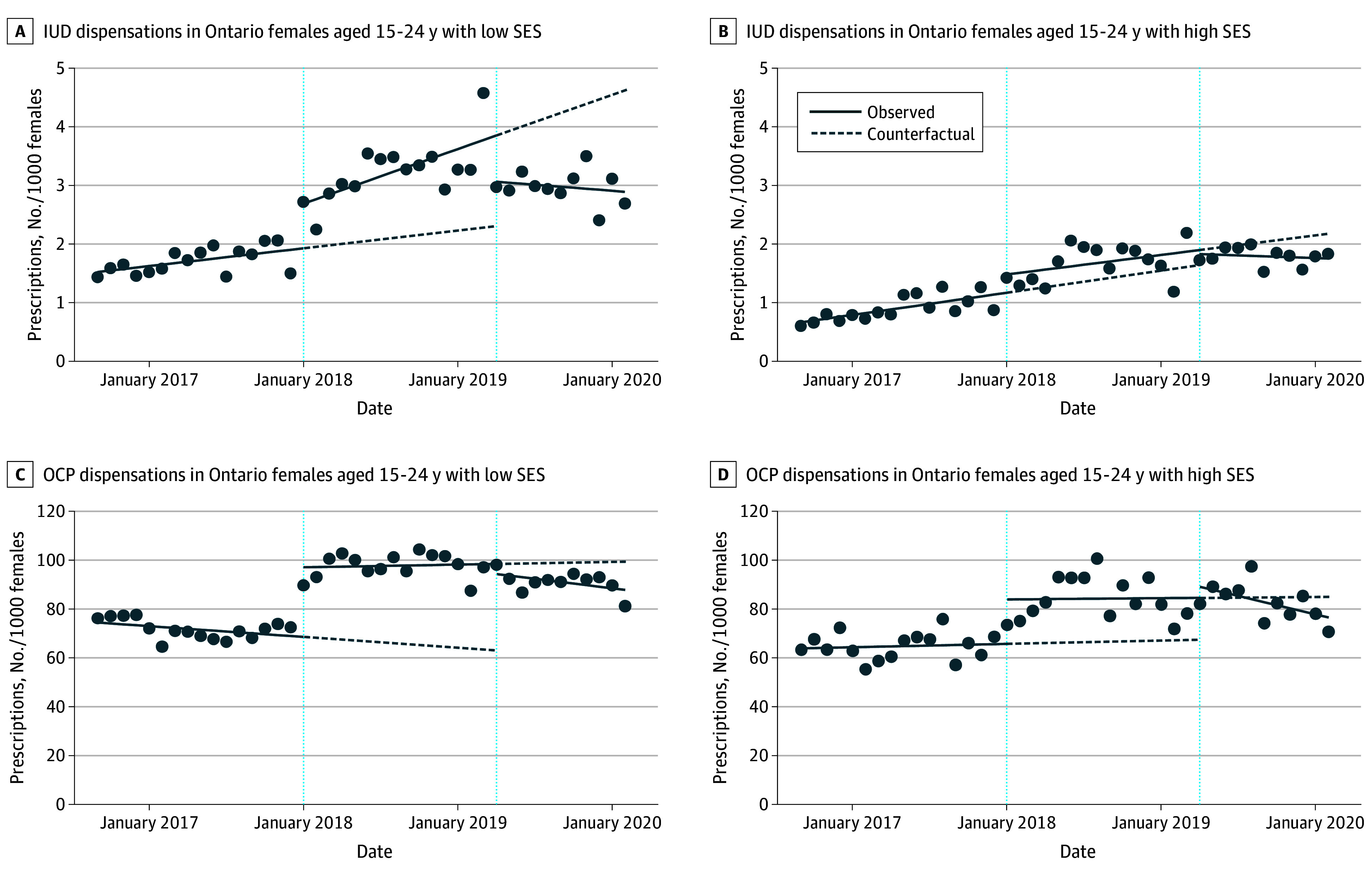
Single Interrupted Time-Series Analysis of Intrauterine Device (IUD) and Oral Contraceptive Pill (OCP) Dispensation Rate in Ontario Females Aged 15 to 24 by Socioeconomic Status (SES)

At the introduction of OHIP+, the level of IUD dispensations increased by 0.76 per 1000 (95% CI, 0.31-1.21) in the low-SES group vs 0.31 per 1000 (95% CI, −0.02 to 0.65) in the high-SES group. Monthly IUD dispensations increased by 0.08 (95% CI, 0.02-0.14) vs 0.03 (95% CI, −0.01 to 0.07) per 1000 after OHIP+ in low- and high-SES groups, respectively. At the introduction of OHIP−, the level of IUD dispensations decreased by 0.79 per 1000 (95% CI, −1.42 to −0.16) and 0.07 per 1000 (95% CI, −0.50 to 0.37) in the low- vs high-SES groups. Monthly IUD dispensations decreased by 0.02 per 1000 (95% CI, −0.07 to 0.03) vs 0.01 per 1000 (95% CI, −0.03 to 0.01) after OHIP− in low- vs high-SES groups, respectively (Figure 3A and B). All quantitative results of the single-group ITS analyses stratified by area-level SES are presented in eTable 3 in Supplement 1.

At the introduction of OHIP+, the level of OCP dispensations increased by 28.5 (95% CI, 21.9-35.2) vs 18.2 (95% CI, 7.51-29.0) per 1000 in the groups with low vs high SES. Monthly OCP dispensations increased by 0.09 (95% CI, −0.65 to 0.83) and 0.04 (95% CI, −1.04 to 1.12) per 1000 after OHIP+ for low and high SES, respectively. At the introduction of OHIP−, the level of OCP dispensations decreased by 4.14 (95% CI, −12.4 to 4.10) vs 0.07 (95% CI, −0.50 to 0.37) per 1000 in the groups with low vs high SES. Monthly OCP dispensations decreased by 0.65 (95% CI, −1.55 to 0.25) vs 1.26 (95% CI, −2.30 to −0.23) per 1000 after OHIP− in groups with low vs high SES, respectively (Figure 3C and D).

**Figure 4.**
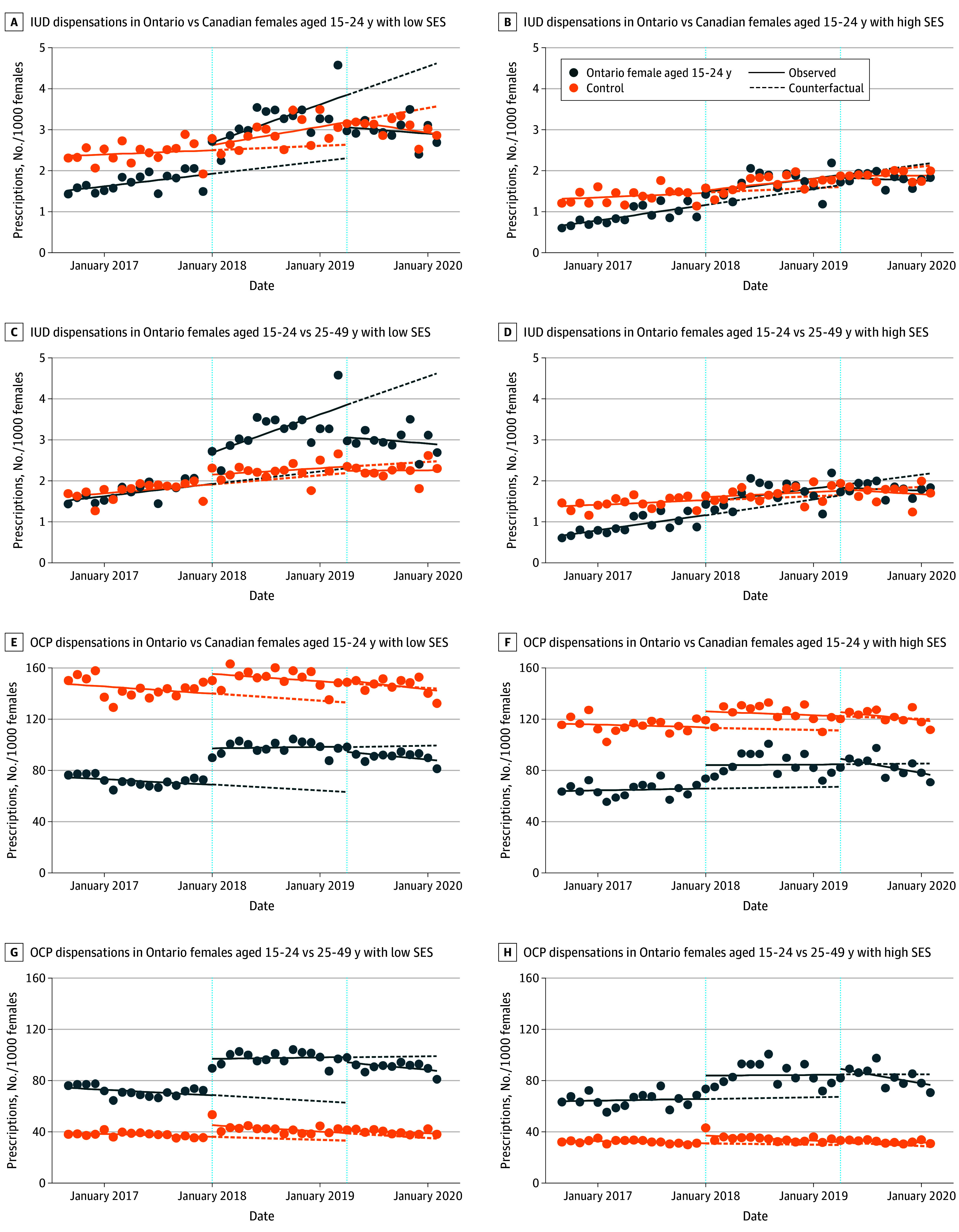
Controlled Interrupted Time-Series Analysis of Intrauterine Device (IUD) and Oral Contraceptive Pill (OCP) Dispensation Rate in Ontario Females Aged 15 to 24 Years by Socioeconomic Status (SES)

In the group with low SES, there was an increase in the level of IUD and OCP dispensations in Ontario vs Canadian females aged 15 to 24 years after OHIP+ by 0.64 (95% CI, 0.02-1.26) and 13.2 (95% CI, 1.33-25.0) per 1000, respectively. After OHIP−, there was a decrease in the level of IUD and OCP dispensations by 0.82 (95% CI, −1.55 to −0.09) and 6.38 (95% CI, −19.2 to 6.47) per 1000, respectively, in Ontario vs Canadian females aged 15 to 24 years. There were no statistically significant changes in the use of IUDs or OCP dispensations vs either control group in the group with high SES (Figure 4). All quantitative results of CITS analyses stratified by SES are presented in eTable 4 in Supplement 1.

Results of the age-stratified analysis (eTables 5-9 in Supplement 1) showed that the level increase in IUD dispensations was numerically greater in females aged 20 to 24 years vs those aged 15 to 19 years when compared with age-matched controls (eFigure in Supplement 1). Results of the sensitivity analyses are presented in eTables 10 to 13 in Supplement 1 and showed that associations between OHIP+/− and dispensation levels and trends were consistent with our primary analyses.

## Discussion

Results from this ITS analysis showed statistically significant and clinically meaningful increases in IUD and OCP dispensations associated with the implementation of OHIP+. For the most part, these results remained consistent after introducing a control group. Although point estimates suggested reduced dispensation volumes after OHIP−, these were generally not significant, with wide CIs in models with controls. We also found a differential association between OHIP+/− and dispensations after stratifying our results by SES, with consistently greater increases in prescriptions dispensed from pharmacies in low-SES areas after OHIP+, a phenomenon that was even more pronounced for IUDs after the inclusion of control groups.

Results of this study add to previous research examining the impact of drug cost-sharing policies on prescription drug utilization. This study reported trends consistent with previously published literature examining the impact of OHIP+ and OHIP−.^20,21,22,23^ Antoniou et al^20,21^ showed an immediate increase in the monthly rate of dispensations of stimulants and benzodiazepines of 0.05 and 0.01 per 1000 individuals, respectively, after OHIP+ and a weaker association between dispensations after OHIP−. Previous changes reported in these studies were quantitatively less than what has been observed in this study on contraceptive dispensations, which reported an immediate increase of 0.50 and 22.33 per 1000 for IUDs and OCPs, respectively, after OHIP+, and a slight reduction in dispensations after OHIP−.

Previous research has reported increased IUD utilization after expanded drug insurance or the provision of free contraception.^6,7,8,9,26,55,56,57,58,59,60^ Results of the Michigan Contraceptive Access, Research, and Evaluation Study (M-CARES) analysis showed that free contraception was associated with a 40% increase in the use of any birth control method and a 324% increased likelihood of choosing long-acting reversible contraceptives (LARCs).^60^ Results of the Contraceptive CHOICE project in St Louis, Missouri, reported that approximately 70% of adolescents who were given standardized counseling and free access to all contraceptives chose a LARC, with the majority of 18- to 20-year-olds selecting an IUD.^6,7^ These results are aligned with the Colorado Family Planning Initiative, which provided LARCs to low-income female individuals at low or no cost; after 6 years of implementation, 31% of clients were LARC users, the highest rate of any state in the US.^9^ Moreover, the introduction of 1 free LARC was associated with a 2.2-fold increase in LARCs dispensed in Finland,^8^ and approximately half of sexually active Irish university students reported that they would change to a LARC if it were free.^58^ These data demonstrate that the relatively high costs of effective contraceptive methods can be a major barrier for young females.

Although the expansion of drug insurance has consistently been associated with increases in prescription drug use, the differences based on SES has been less ascertained.^1,2,39,40,60,61,62,63^ Previous studies have reported large SES gradients in drug insurance across Canada and in Ontario. These results have shown that individuals with low SES are more likely to report public or no drug coverage^61^; however, to-date, there are limited data available on how household income may impact choice of contraceptive methods among Canadian youth.^60,64^ Our results suggest a stronger association between OHIP+/− and contraceptive dispensations among females who obtained their prescription from pharmacies located in lower SES areas. This is aligned with previous research which has shown that increased dispensations after OHIP+ were greater in females, low SES groups, and individuals aged 20 to 24 years.^20,22^ Literature regarding the impact of SES has suggested that adolescent females with lower SES engaged in sexual activity earlier,^65^ and may therefore need better access to contraception.

### Limitations

This study has some limitations. First, we did not have access to individual-level data and were therefore not able to estimate utilization. We have also assumed consistent volumes dispensed per policy period; however, OCP users may have increased the volume of OCPs dispensed at one time after OHIP+, which may lead to overestimated treatment effects. Second, we assumed ordinary least-squares regression and linear trends throughout the model because these models are more flexible and broadly applicable for ITS analysis; however, failing to account for nonlinear trends can lead to overestimated or underestimated treatment effects.^66^ Third, dispensations stratified by SES quintiles were estimated by dividing the linearly interpolated annual population into 5, which may lead to overestimates or underestimates and could bias the differential trends we observed after stratifying by SES.

## Conclusions

Numerous studies have reported financial barriers associated with accessing effective contraception.^26^ In order to improve access to effective contraceptives, medical societies have been calling for universal and confidential access to contraception, especially in individuals younger than the age of 25 years.^14,67^ The introduction of OHIP+ and OHIP− provides a unique opportunity to examine the impact of drug insurance and confidentiality on contraceptive dispensations in youth. Results of this cohort study reveal that there were greater changes in dispensation associated with universal coverage under OHIP+ than with OHIP−, which provided needs-based coverage for individuals without drug insurance and compromised confidentiality for privately covered youth. These results suggest supporting universal contraceptive coverage for youth. Future analyses should evaluate the subsequent impacts of increased access to contraception, including effects on abortions and teenage pregnancy.

## Notes

Supplement 1. **eFigure.** Controlled ITS of Prescription Contraceptives Dispensation Rate in 15- to 19-Year-Olds and 20- to 24-Year-Olds **eTable 1.** Single-Group Interrupted Time Series of Prescription Contraceptives Dispensed per 1000 Females Before and After Implementation of OHIP+ and OHIP− in Ontario Youth Under the Age of 25 Years **eTable 2.** Controlled Interrupted Time Series of Prescription Contraceptives Dispensed per 1000 Females Before and After Implementation of OHIP+ and OHIP− for Ontario Youth Under the Age of 25 Years vs Control Groups **eTable 3.** Single-Group Interrupted Time Series of Prescription Contraceptives Dispensed per 1000 Females Before and After Implementation of OHIP+ and OHIP− in Ontario Females 15- to 24-Years-Old Stratified by SES **eTable 4.** Controlled Interrupted Time Series of Prescription Contraceptives Dispensed per 1000 Females Before and After Implementation of OHIP+ and OHIP− for Ontario Youth Under the Age of 25 vs Control Groups Stratified by SES **eTable 5.** Single-Group Interrupted Time Series of Prescription Contraceptives Dispensed per 1000 Females Before and After Implementation of OHIP+ and OHIP− in Ontario Youth Aged 15- to 19- and 20- to 24-Years-Old **eTable 6.** Controlled Interrupted Time Series of Prescription Contraceptives Dispensed per 1000 Females Before and After Implementation of OHIP+ and OHIP− for Ontario Youth Aged 15- to 19- and 20- to 24-Years-Old vs Age-Matched Canadian Cohorts **eTable 7.** Single-Group Interrupted Time Series of Prescription Contraceptives Dispensed per 1000 Females Before and After Implementation of OHIP+ and OHIP− in Ontario Females 15- to 19-Years-Old Stratified by SES **eTable 8.** Single-Group Interrupted Time Series of Prescription Contraceptives Dispensed per 1000 Females Before and After Implementation of OHIP+ and OHIP− in Ontario Females 20- to 24-Years-Old Stratified by SES **eTable 9.** Controlled Interrupted Time Series of Prescription Contraceptives Dispensed per 1000 Females Before and After Implementation of OHIP+ and OHIP− for Ontario Youth Aged 15- to 19- and 20- to 24-Years-Old Age-Matched Canadian Cohorts and Stratified by SES **eTable 10.** Sensitivity Analysis—Single-Group Interrupted Time Series of Prescription Contraceptives Dispensed per 1000 Females Before and After Implementation of OHIP+ and OHIP− in Ontario Youth Under the Age of 25, Using February 2018 and May 2019 as Intervention Dates **eTable 11.** Sensitivity Analysis—Controlled Interrupted Time Series of Prescription Contraceptives Dispensed per 1000 Females Before and After Implementation of OHIP+ and OHIP− for Ontario Youth Under the Age of 25 vs Control Groups, Using February 2018 and May 2019 as Intervention Dates **eTable 12.** Sensitivity Analysis—Single-Group Interrupted Time Series of Prescription Contraceptives Dispensed per 1000 Females Before and After Implementation of OHIP+ and OHIP− in Ontario Females 15- to 24-Years-Old Stratified by SES, Using February 2018 and May 2019 as Intervention Dates **eTable 13.** Sensitivity Analysis—Controlled Interrupted Time Series of Prescription Contraceptives Dispensed per 1000 Females Before and After Implementation of OHIP+ and OHIP− for Ontario Youth Under the Age of 25 vs Control Groups Stratified by SES, Using February 2018 and May 2019 as Intervention Dates

Supplement 2.
Data Sharing Statement.
