# Supplementary material for: Universal Pharmacare and Contraceptive Dispensations Among Youth
Source: JAMA Pediatr. 2025 Aug 18;179(10):1090–9. doi: 10.1001/jamapediatrics.2025.2585 (PMC12362276; doi:10.1001/jamapediatrics.2025.2585)
Supplement: Supplement 1. — eFigure. Controlled ITS of Prescription Contraceptives Dispensation Rate in 15- to 19-Year-Olds and 20- to 24-Year-Olds eTable 1. Single-Group Interrupted Time Series of Prescription Contraceptives Dispensed per 1000 Females Before and After Implementation of OHIP+ and OHIP− in Ontario Youth Under the Age of 25 Years eTable 2. Controlled Interrupted Time Series of Prescription Contraceptives Dispensed per 1000 Females Before and After Implementation of OHIP+ and OHIP− for Ontario Youth Under the Age of 25 Years vs Control Groups eTable 3. Single-Group Interrupted Time Series of Prescription Contraceptives Dispensed per 1000 Females Before and After Implementation of OHIP+ and OHIP− in Ontario Females 15- to 24-Years-Old Stratified by SES eTable 4. Controlled Interrupted Time Series of Prescription Contraceptives Dispensed per 1000 Females Before and After Implementation of OHIP+ and OHIP− for Ontario Youth Under the Age of 25 vs Control Groups Stratified by SES eTable 5. Single-Group Interrupted Time Series of Prescription Contraceptives Dispensed per 1000 Females Before and After Implementation of OHIP+ and OHIP− in Ontario Youth Aged 15- to 19- and 20- to 24-Years-Old eTable 6. Controlled Interrupted Time Series of Prescription Contraceptives Dispensed per 1000 Females Before and After Implementation of OHIP+ and OHIP− for Ontario Youth Aged 15- to 19- and 20- to 24-Years-Old vs Age-Matched Canadian Cohorts eTable 7. Single-Group Interrupted Time Series of Prescription Contraceptives Dispensed per 1000 Females Before and After Implementation of OHIP+ and OHIP− in Ontario Females 15- to 19-Years-Old Stratified by SES eTable 8. Single-Group Interrupted Time Series of Prescription Contraceptives Dispensed per 1000 Females Before and After Implementation of OHIP+ and OHIP− in Ontario Females 20- to 24-Years-Old Stratified by SES eTable 9. Controlled Interrupted Time Series of Prescription Contraceptives Dispensed per 1000 Females Before and After Implementation of OHI [file jamapediatr-e252585-s001.pdf]

Supplementary Online Content

Downey AK, Hanna SE, Levine MA, Schummers L, Guindon GE. Universal pharmacare and contraceptive dispensations among youth. *JAMA Pediatr*. Published online August 18, 2025. doi:10.1001/jamapediatrics.2025.2585

eFigure. Controlled ITS of Prescription Contraceptives Dispensation Rate in 15- to 19-Year-Olds and 20- to 24-Year-Olds

eTable 1. Single-Group Interrupted Time Series of Prescription Contraceptives Dispensed per 1000 Females Before and After Implementation of OHIP+ and OHIP− in Ontario Youth Under the Age of 25 Years

eTable 2. Controlled Interrupted Time Series of Prescription Contraceptives Dispensed per 1000 Females Before and After Implementation of OHIP+ and OHIP− for Ontario Youth Under the Age of 25 Years vs Control Groups

eTable 3. Single-Group Interrupted Time Series of Prescription Contraceptives Dispensed per 1000 Females Before and After Implementation of OHIP+ and OHIP− in Ontario Females 15- to 24-Years-Old Stratified by SES

eTable 4. Controlled Interrupted Time Series of Prescription Contraceptives Dispensed per 1000 Females Before and After Implementation of OHIP+ and OHIP− for Ontario Youth Under the Age of 25 vs Control Groups Stratified by SES

eTable 5. Single-Group Interrupted Time Series of Prescription Contraceptives Dispensed per 1000 Females Before and After Implementation of OHIP+ and OHIP− in Ontario Youth Aged 15- to 19- and 20- to 24-Years-Old

eTable 6. Controlled Interrupted Time Series of Prescription Contraceptives Dispensed per 1000 Females Before and After Implementation of OHIP+ and OHIP− for Ontario Youth Aged 15- to 19- and 20- to 24-Years-Old vs Age-Matched Canadian Cohorts

eTable 7. Single-Group Interrupted Time Series of Prescription Contraceptives Dispensed per 1000 Females Before and After Implementation of OHIP+ and OHIP− in Ontario Females 15- to 19-Years-Old Stratified by SES

eTable 8. Single-Group Interrupted Time Series of Prescription Contraceptives Dispensed per 1000 Females Before and After Implementation of OHIP+ and OHIP− in Ontario Females 20- to 24-Years-Old Stratified by SES

eTable 9. Controlled Interrupted Time Series of Prescription Contraceptives Dispensed per 1000 Females Before and After Implementation of OHIP+ and OHIP− for Ontario Youth Aged 15- to 19- and 20- to 24-Years-Old Age-Matched Canadian Cohorts and Stratified by SES

© 2025 Downey AK et al. *JAMA Pediatrics*.

eTable 10. Sensitivity Analysis—Single-Group Interrupted Time Series of Prescription Contraceptives Dispensed per 1000 Females Before and After Implementation of OHIP+ and OHIP− in Ontario Youth Under the Age of 25, Using February 2018 and May 2019 as Intervention Dates

eTable 11. Sensitivity Analysis—Controlled Interrupted Time Series of Prescription Contraceptives Dispensed per 1000 Females Before and After Implementation of OHIP+ and OHIP− for Ontario Youth Under the Age of 25 vs Control Groups, Using February 2018 and May 2019 as Intervention Dates

eTable 12. Sensitivity Analysis—Single-Group Interrupted Time Series of Prescription Contraceptives Dispensed per 1000 Females Before and After Implementation of OHIP+ and OHIP− in Ontario Females 15- to 24-Years-Old Stratified by SES, Using February 2018 and May 2019 as Intervention Dates

eTable 13. Sensitivity Analysis—Controlled Interrupted Time Series of Prescription Contraceptives Dispensed per 1000 Females Before and After Implementation of OHIP+ and OHIP− for Ontario Youth Under the Age of 25 vs Control Groups Stratified by SES, Using February 2018 and May 2019 as Intervention Dates

This supplementary material has been provided by the authors to give readers additional information about their work.

© 2025 Downey AK et al. *JAMA Pediatrics*.

eFigure. Controlled interrupted time series of prescription contraceptives dispensed per 1000 females in (a) 15-19 year-olds and (b) 20-24 year-olds before and after the introduction of OHIP+ in January 2018 and OHIP- in April 2019

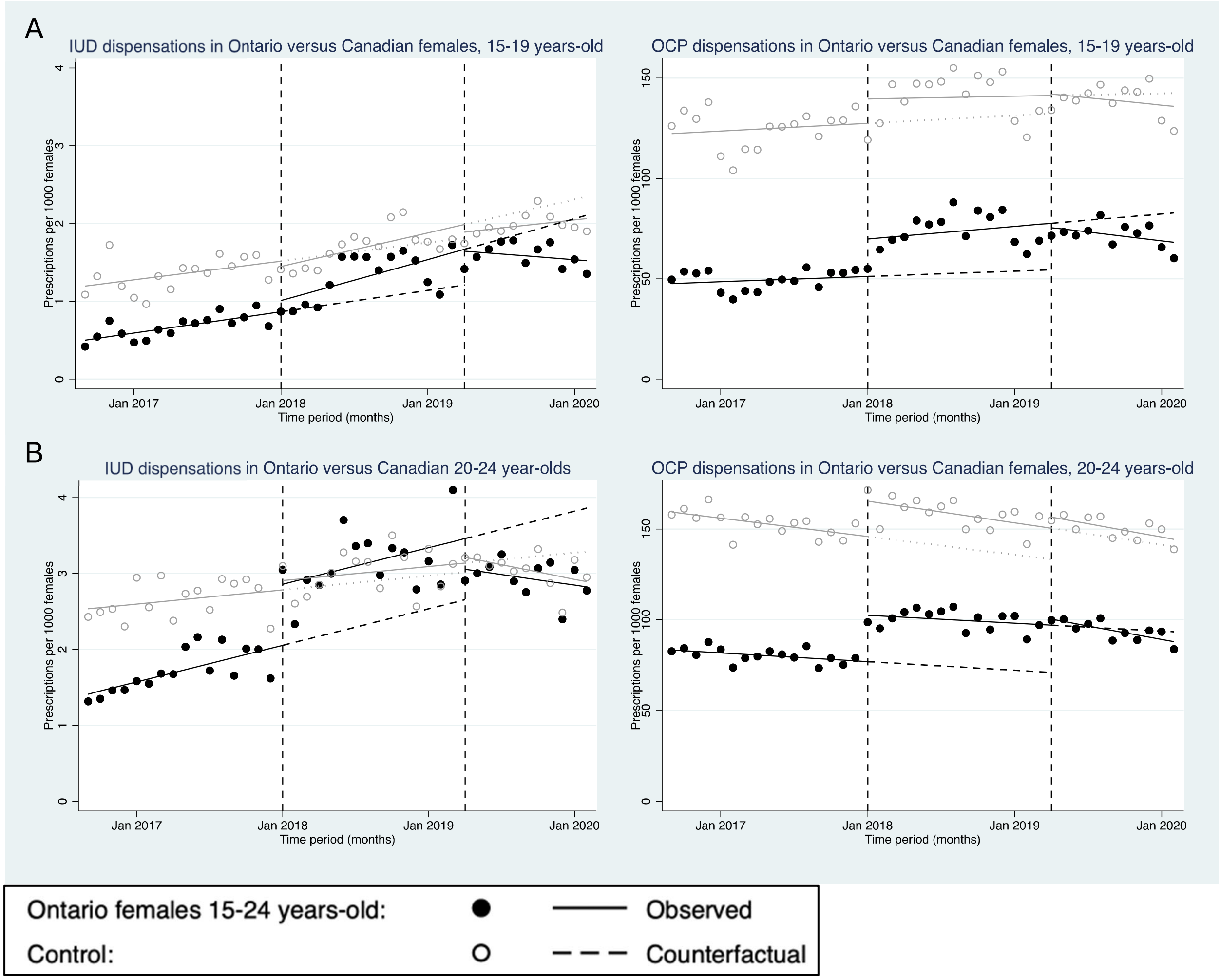

© 2025 Downey AK et al. *JAMA Pediatrics*.

eTable 1. Single-group interrupted time series of prescription contraceptives dispensed per 1000 females before and after implementation of OHIP+ and OHIP- in Ontario youth under the age of 25							
**Pre-policy (Sep 2016 - Dec 2017)**		**OHIP+ (Jan 2018 - Mar 2019)**			**OHIP- (Apr 2019 - Feb 2020)**		
Starting level	Monthly change	Level change	Relative monthly change	Monthly change	Level change	Relative monthly change	Monthly change
Dispensations (95% CI)	Dispensations (95% CI)	Dispensations (95% CI)	Dispensations (95% CI)	Dispensations (95% CI)	Dispensations (95% CI)	Dispensations (95% CI)	Dispensations (95% CI)
**Intrauterine devices, Ontario 15-24 years-old (lag 0)**							
0.98 (0.90, 1.06)	0.03 (0.02, 0.05)	0.50 (0.27, 0.73)	0.01 (-0.03, 0.05)	0.04 (0.01, 0.07)	-0.23 (-0.50, 0.04)	-0.06 (-0.10, -0.02)	-0.02 (-0.04, 0.00)
**Intrauterine devices, Canada[a] 15-24 years-old (lag 0)**							
1.91 (1.74, 2.08)	0.02 (-0.01, 0.04)	0.03 (-0.26, 0.32)	0.01 (-0.02, 0.04)	0.03 (0.00, 0.05)	-0.01 (-0.26, 0.24)	-0.03 (-0.06, -0.00)	-0.01 (-0.03, 0.01)
**Intrauterine devices, Ontario 25-49 years-old (lag 3)**							
1.48 (1.37, 1.60)	0.01 (-0.00, 0.03)	0.19 (-0.05, 0.33)	-0.00 (-0.02, 0.02)	0.01 (-0.00, 0.02)	0.01 (-0.13, 0.15)	-0.02 (-0.04, 0.01)	-0.01 (-0.03, 0.02)
**Oral contraceptives, Ontario 15-24 years-old (lag 0)**							
66.54 (62.97, 70.12)	-0.10 (-0.45, 0.25)	22.33 (14.81, 29.84)	0.16 (-0.78, 1.08)	0.06 (-0.80, 0.91)	0.74 (-7.84, 9.32)	-1.05 (-2.18, 0.07)	-1.00 (-1.73, -0.27)
**Oral contraceptives, Canada[a] 15-24 years-old (lag 0)**							
142.03 (134.69, 149.36)	-0.30 (-0.99, 0.38)	16.07 (5.91, 26.22)	-0.18 (-1.46, 1.11)	-0.48 (-1.57, 0.61)	3.60 (-7.36, 14.56)	-0.46 (-2.05, 1.14)	-0.93 (-2.10, 0.23)
**Oral contraceptives, Ontario 25-49 years-old (lag 0)**							
36.37 (35.01, 37.73)	-0.15 (-0.28, -0.03)	7.57 (3.07, 12.07)	-0.23 (-0.73, 0.28)	-0.38 (-0.87, 0.11)	1.97 (-1.52, 5.47)	0.13 (-0.42, 0.69)	-0.24 (-0.50, 0.01)

[a] *Refers to prescriptions dispensed in females from all Canadian provinces, excluding Ontario*

© 2025 Downey AK et al. *JAMA Pediatrics*.

eTable 2. Controlled interrupted time series of prescription contraceptives dispensed per 1000 females before and after implementation of OHIP+ and OHIP- for Ontario youth under the age of 25 versus control groups					
**Pre-policy (Sep 2016 - Dec 2017)**		**OHIP+ (Jan 2018 – Mar 2019)**		**OHIP- (Apr 2019 – Feb 2020)**	
Difference in starting level	Difference in monthly change	Difference in level change	Difference in monthly change	Difference in level change	Difference in monthly change
Dispensations (95% CI)	Dispensations (95% CI)	Dispensations (95% CI)	Dispensations (95% CI)	Dispensations (95% CI)	Dispensations (95% CI)
**Intrauterine devices, control: Canada[a] 15-24 year-olds (lag 0)**					
-0.93 (-1.12, -0.74)	0.01 (-0.01, 0.04)	0.47 (0.02, 0.91)	0.00 (-0.05, 0.06)	-0.22 (-0.72, 0.28)	-0.03 (-0.09, 0.03)
**Intrauterine devices, control: Ontario 25-49 year-olds (lag 0)**					
-0.50 (-0.69, -0.31)	0.02 (-0.01, 0.05)	0.31 (-0.10, 0.73)	0.01 (-0.04, 0.07)	-0.24 (-0.75, 0.27)	-0.04 (-0.11, 0.02)
**Oral contraceptives, control: Canada[a] 15-24 year-olds (lag 0)**					
-75.49 (-83.51, -67.47)	0.20 (-0.56, 0.96)	6.26 (-6.16, 18.68)	0.33 (-1.23, 1.89)	-2.86 (-16.54, 10.81)	-0.60 (-2.51, 1.32)
**Oral contraceptives, control: Ontario 25-49 year-olds (lag 0)**					
30.17 (26.41, 33.93)	0.05 (-0.32, 0.42)	14.76 (6.15, 23.37)	0.38 (-0.65, 1.42)	-1.23 (-10.34, 7.87)	-1.19 (-2.42, 0.04)

[a]*Refers to prescriptions dispensed in females from all Canadian provinces, excluding Ontario*

© 2025 Downey AK et al. *JAMA Pediatrics*.

eTable 3. Single-group interrupted time series of prescription contraceptives dispensed per 1000 females before and after implementation of OHIP+ and OHIP- in Ontario females 15-24 years-old stratified by SES

Pre-policy (Sep 2016 - Dec 2017)		OHIP+ (Jan 2018 - Mar 2019)			OHIP- (Apr 2019 - Feb 2020)		
Starting level	Monthly change	Level change	Relative monthly change	Monthly change	Level change	Relative monthly change	Monthly change
Dispensations (95% CI)	Dispensations (95% CI)	Dispensations (95% CI)	Dispensations (95% CI)	Dispensations (95% CI)	Dispensations (95% CI)	Dispensations (95% CI)	Dispensations (95% CI)
**Intrauterine devices, low socioeconomic status (lag 0)**							
1.52 (1.38, 1.66)	0.03 (0.00, 0.05)	0.76 (0.31, 1.21)	0.05 (-0.01, 0.12)	0.08 (0.02, 0.14)	-0.79 (-1.42, -0.16)	-0.09 (-0.17, -0.02)	-0.02 (-0.07, 0.03)
**Intrauterine devices, low-middle socioeconomic status (lag 0)**							
1.24 (1.09, 1.39)	0.03 (0.01, 0.05)	0.80 (0.38, 1.23)	0.01 (-0.04, 0.05)	0.04 (-0.01, 0.08)	-0.19 (-0.74, 0.36)	-0.08 (-0.15, -0.00)	-0.04 (-0.10, 0.02)
**Intrauterine devices, middle socioeconomic status (lag 0)**							
0.76 (0.65, 0.88)	0.03 (0.02, 0.05)	0.21 (-0.14, 0.56)	0.00 (-0.04, 0.05)	0.04 (0.00, 0.08)	-0.06 (-0.45, 0.33)	-0.06 (-0.11, -0.02)	-0.02 (-0.05, 0.00)
**Intrauterine devices, middle-high socioeconomic status (lag 0)**							
0.72 (0.61, 0.83)	0.04 (0.02, 0.05)	0.40 (0.06, 0.74)	-0.01 (-0.05, 0.03)	0.03 (-0.01, 0.07)	-0.03 (-0.47, 0.41)	-0.03 (-0.09, 0.03)	0.00 (-0.04, 0.04)
**Intrauterine devices, high socioeconomic status (lag 0)**							
0.67 (0.57, 0.76)	0.03 (0.01, 0.05)	0.31 (-0.02, 0.65)	-0.00 (-0.05, 0.04)	0.03 (-0.01, 0.07)	-0.07 (-0.50, 0.37)	-0.04 (-0.08, 0.01)	-0.01 (-0.03, 0.01)
**Oral contraceptives, low socioeconomic status (lag 1)**							
74.53 (70.22, 78.83)	-0.37 (-0.85, 0.11)	28.53 (20.50, 36.55)	0.46 (-0.43 1.35)	0.09 (-0.71, 0.89)	-4.14 (-12.81, 4.53)	-0.74 (-1.92, 0.43)	-0.65 (-1.53, 0.22)
**Oral contraceptives, low-middle socioeconomic status (lag 0)**							
70.09 (66.68, 73.49)	-0.16 (-0.48, 0.16)	25.63 (18.41, 32.85)	0.16 (-0.73, 1.06)	0.01 (-0.83, 0.84)	-0.04 (-8.07, 8.00)	-0.93 (-2.01, 0.14)	-0.93 (-1.61, -0.25)
**Oral contraceptives, middle socioeconomic status (lag 0)**							
60.19 (56.68, 63.71)	-0.06 (-0.41, 0.29)	19.26 (11.13, 27.38)	0.06 (-0.90, 1.03)	0.00 (-0.90, 0.90)	2.40 (-7.32, 12.12)	-1.10 (-2.35, 0.15)	-1.09 (-1.96, -0.23)
**Oral contraceptives, middle-high socioeconomic status (lag 0)**							
63.92 (60.25, 67.60)	-0.03 (-0.41, 0.36)	19.98 (12.33, 27.63)	0.16 (-0.75, 1.08)	0.14 (-0.69, 0.97)	0.94 (-7.54, 9.43)	-1.19 (-2.30, -0.08)	-1.05 (-1.79, -0.32)
**Oral contraceptives, high socioeconomic status (lag 0)**							
63.97 (59.45, 68.51)	0.11 (-0.41, 0.64)	18.23 (7.51, 28.96)	-0.07 (-1.27, 1.13)	0.04 (-1.04, 1.12)	4.54 (-7.43, 16.51)	-1.30 (-2.80, 0.19)	-1.26 (-2.30, -0.23)

© 2025 Downey AK et al. *JAMA Pediatrics*.

eTable 4. Controlled interrupted time series of prescription contraceptives dispensed per 1000 females before and after implementation of OHIP+ and OHIP- for Ontario youth under the age of 25 versus control groups stratified by SES					
**Pre-policy (Sep 2016 – Dec 2017)**		**OHIP+ (Jan 2018 – Mar 2019)**		**OHIP- (Apr 2019 – Feb 2020)**	
Difference in starting level	Difference in monthly change	Difference in level change	Difference in monthly change	Difference in level change	Difference in monthly change
Dispensations (95% CI)	Dispensations (95% CI)	Dispensations (95% CI)	Dispensations (95% CI)	Dispensations (95% CI)	Dispensations (95% CI)
**Intrauterine devices – low socioeconomic status, control: Canada[a] 15-24 year-olds (lag 0)**					
-0.84 (-1.10, -0.59)	0.02 (-0.02, 0.06)	0.64 (0.02, 1.26)	0.02 (-0.06, 0.10)	-0.82 (-1.55, -0.09)	-0.02 (-0.11, 0.06)
**Intrauterine devices – low socioeconomic status, control: Ontario 25-49 year-olds (lag 0)**					
-0.10 (-0.33, 0.13)	0.01 (-0.03, 0.04)	0.52 (-0.00, 1.05)	0.06 (-0.02, 0.13)	-0.68 (-1.39, 0.02)	-0.08 (-0.17, 0.01)
**Intrauterine devices – high socioeconomic status, control: Canada[a] 15-24 year-olds (lag 0)**					
-0.65 (-0.83, -0.48)	0.02 (-0.00, 0.05)	0.25 (-0.17, 0.67)	-0.02 (-0.07, 0.03)	-0.07 (-0.53, 0.40)	-0.01 (-0.06, 0.04)
**Intrauterine devices – high socioeconomic status, control: Ontario 25-49 year-olds (lag 0)**					
-0.71 (-0.87, -0.55)	0.02 (-0.00, 0.05)	0.24 (-0.15, 0.63)	-0.01 (-0.06, 0.05)	-0.10 (-0.62, 0.42)	-0.01 (-0.08, 0.05)
**Oral contraceptives – low socioeconomic status, control: Canada[a] 15-24 year-olds (lag 0)**					
-72.78 (-81.44, -64.11)	0.10 (-0.75, 0.95)	13.17 (1.33, 25.00)	0.47 (-1.03, 1.97)	-6.38 (-19.22, 6.47)	-0.39 (-2.33, 1.54)
**Oral contraceptives – low socioeconomic status, control: Ontario 25-49 year-olds (lag 0)**					
35.20 (31.34, 39.06)	-0.18 (-0.59, 0.23)	19.48 (11.24, 27.71)	0.69 (-0.31, 1.69)	-6.43 (-15.51, 2.66)	-0.90 (-2.21, 0.41)
**Oral contraceptives – high socioeconomic status, control: Canada[a] 15-24 year-olds (lag 3)**					
-52.43 (-59.50, -45.35)	0.29 (-0.33, 0.90)	5.90 (-11.01, 22.80)	0.00 (-1.70, 1.70)	1.44 (-10.80, 13.68)	-0.85 (-3.12, 1.42)
**Oral contraceptives – high socioeconomic status, control: Ontario 25-49 year-olds (lag 0)**					
30.61 (25.98, 35.24)	0.25 (-0.28, 0.78)	12.15 (0.95, 23.35)	0.13 (-1.13, 1.39)	3.20 (-8.92, 15.31)	-1.46 (-2.99, 0.08)

[a]*Refers to prescriptions dispensed in females from all Canadian provinces, excluding Ontario*

© 2025 Downey AK et al. *JAMA Pediatrics*.

**eTable 5. Single-group interrupted time series of prescription contraceptives dispensed per 1000 females before and after implementation of OHIP+ and OHIP- in Ontario youth aged 15-19 and 20-24 years-old**							
**Pre-policy (Sep 2016 - Dec 2017)**		**OHIP+ (Jan 2018 - Mar 2019)**			**OHIP- (Apr 2019 - Feb 2020)**		
Starting level	Monthly change	Level change	Relative monthly change	Monthly change	Level change	Relative monthly change	Monthly change
Dispensations (95% CI)	Dispensations (95% CI)	Dispensations (95% CI)	Dispensations (95% CI)	Dispensations (95% CI)	Dispensations (95% CI)	Dispensations (95% CI)	Dispensations (95% CI)
**Intrauterine devices, Ontario 15-19 years-old (lag 0)**							
0.50 (0.39, 0.61)	0.02 (0.01, 0.04)	0.14 (-0.10, 0.38)	0.02 (-0.01, 0.05)	0.04 (0.01, 0.07)	-0.02 (-0.38, 0.34)	-0.06 (-0.10, -0.01)	-0.01 (-0.04, 0.02)
**Intrauterine devices, Ontario 20-24 years-old (lag 0)**							
1.41 (1.29, 1.53)	0.04 (0.02, 0.06)	0.81 (0.34, 1.27)	-0.00 (-0.06, 0.06)	0.04 (-0.01, 0.10)	-0.40 (-0.96, 0.16)	-0.06 (-0.13, 0.00)	-0.02 (-0.06, 0.01)
**Intrauterine devices, Canada[a] 15-19 years-old (lag 1)**							
1.20 (0.94, 1.45)	0.02 (-0.00, 0.04)	-0.07 (-0.29, 0.15)	0.02 (-0.02, 0.05)	0.04 (0.01, 0.06)	-0.10 (-0.38, 0.19)	-0.02 (-0.06, 0.02)	0.02 (-0.01, 0.04)
**Intrauterine devices, Canada[a] 20-24 years-old (lag 1)**							
2.53 (2.36, 2.71)	0.02 (-0.01, 0.04)	0.12 (-0.29, 0.54)	-0.00 (-0.03, 0.03)	0.02 (-0.01, 0.04)	0.07 (-0.16, 0.31)	-0.05 (-0.09, -0.01)	-0.03 (-0.06, -0.00)
**Oral contraceptives, Ontario 15-19 years-old (lag 0)**							
47.64 (42.68, 52.61)	0.22 (-0.25, 0.69)	18.67 (8.53, 28.82)	0.30 (-1.02, 1.62)	0.52 (-0.72, 1.76)	-2.16 (-14.53, 10.21)	-1.25 (-2.85, 0.34)	-0.73 (1.74, 0.28)
**Oral contraceptives, Ontario 20-24 years-old (lag 0)**							
83.34 (80.27, 86.40)	-0.40 (-0.73, -0.07)	25.51 (19.72, 31.30)	0.04 (-0.65, 0.72)	-0.36 (-0.97, 0.24)	3.27 (-3.10, 9.65)	-0.88 (-1.73, -0.02)	-1.24 (-1.85, -0.63)
**Oral contraceptives, Canada[a] 15-19 years-old (lag 1)**							
122.35 (109.85, 138.84)	0.32 (-0.75, 1.39)	12.17 (-6.26, 30.60)	-0.21 (-2.43, 2.01)	0.11 (-1.94, 2.17)	0.70 (-18.87, 20.28)	-0.72 (-3.54, 2.11)	-0.61 (-2.47, 1.26)
**Oral contraceptives, Canada[a] 20-24 years-old (lag 1)**							
159.28 (154.66, 163.90)	-0.84 (-1.29, -0.39)	19.50 (13.74, 25.26)	-0.16 (-1.00, 0.68)	-1.00 (-1.64, -0.36)	6.16 (-0.40, 12.73)	-0.23 (-1.15, 0.69)	-1.23 (-1.93, -0.52)

[a]*Refers to prescriptions dispensed in females from all Canadian provinces, excluding Ontario*

© 2025 Downey AK et al. *JAMA Pediatrics*.

eTable 6. Controlled interrupted time series of prescription contraceptives dispensed per 1000 females before and after implementation of OHIP+ and OHIP- for Ontario youth aged 15-19 and 20-24 years-old versus age-matched Canadian cohorts					
**Pre-policy (Sep 2016 - Dec 2017)**		**OHIP+ (Jan 2018 – Mar 2019)**		**OHIP- (Apr 2019 – Feb 2020)**	
Difference in starting level	Difference in monthly change	Difference in level change	Difference in monthly change	Difference in level change	Difference in monthly change
Dispensations (95% CI)	Dispensations (95% CI)	Dispensations (95% CI)	Dispensations (95% CI)	Dispensations (95% CI)	Dispensations (95% CI)
**Intrauterine devices 15-19 year-olds, control: Canada[a] 15-19 year-olds (lag 1)**					
-0.70 (-0.97, -0.42)	0.00 (-0.02, 0.03)	0.21 (-0.13, 0.55)	0.01 (-0.05, -0.06)	0.07 (-0.43, 0.57)	-0.04 (-0.10, 0.02)
**Intrauterine devices, 20-24 year-olds, control: Canada[a] 20-24 year-olds (lag 1)**					
-1.12 (-1.34, -0.91)	0.02 (-0.01, 0.06)	0.68 (0.08, 1.29)	0.00 (-0.06, 0.06)	-0.48 (-1.06, 0.10)	-0.02 (-0.08, 0.05)
**Oral contraceptives 15-19 year-olds, control: Canada[a] 15-19 year-olds (lag 5)**					
-74.70 (-87.91, -61.49)	-0.10 (-1.31, 1.11)	6.50 (-18.51, 31.53)	0.51 (-1.89, 2.90)	-2.87 (-21.63, 15.89)	-0.53 (-3.89, 2.83)
**Oral contraceptives 20-24 year-olds, control: Canada[a] 20-24 year-olds (lag 1)**					
-75.94 (-81.34, -70.54)	0.44 (-0.07, 0.95)	6.01 (-2.48, 14.50)	0.19 (-0.88, 1.27)	-2.89 (-11.46, 5.68)	-0.65 (-1.88, 0.58)

[a]*Refers to prescriptions dispensed in females from all Canadian provinces, excluding Ontario*

© 2025 Downey AK et al. *JAMA Pediatrics*.

eTable 7. Single-group interrupted time series of prescription contraceptives dispensed per 1000 females before and after implementation of OHIP+ and OHIP- in Ontario females 15-19 years-old stratified by SES							
**Pre-policy (Sep 2016 - Dec 2017)**		**OHIP+ (Jan 2018 - Mar 2019)**			**OHIP- (Apr 2019 - Feb 2020)**		
Starting level	Monthly change	Level change	Relative monthly change	Monthly change	Level change	Relative monthly change	Monthly change
Dispensations (95% CI)	Dispensations (95% CI)	Dispensations (95% CI)	Dispensations (95% CI)	Dispensations (95% CI)	Dispensations (95% CI)	Dispensations (95% CI)	Dispensations (95% CI)
**Intrauterine devices, low socioeconomic status (lag 0)**							
0.68 (0.48, 0.88)	0.02 (-0.00, 0.05)	0.03 (-0.27, 0.34)	0.04 (0.00, 0.08)	0.07 (0.04, 0.10)	-0.22 (-0.63, 0.18)	-0.07 (-0.13, -0.02)	-0.01 (-0.05, 0.04)
**Intrauterine devices, low-middle socioeconomic status (lag 0)**							
0.73 (0.59, 0.87)	0.02 (0.01, 0.04)	0.37 (0.03, 0.70)	0.02 (-0.02, 0.06)	0.05 (0.01, 0.08)	-0.07 (-0.55, 0.40)	-0.07 (-0.14, -0.01)	-0.03 (-0.08, 0.02)
**Intrauterine devices, middle socioeconomic status (lag 1)**							
0.39 (0.26, 0.52)	0.03 (0.01, 0.04)	0.03 (-0.27, 0.32)	0.01 (-0.03, 0.05)	0.04 (0.00, 0.07)	0.02 (-0.37, 0.40)	-0.04 (-0.09, -0.00)	-0.01 (-0.03, 0.02)
**Intrauterine devices, middle-high socioeconomic status lag 0)**							
0.38 (0.26, 0.49)	0.03 (0.01, 0.04)	0.08 (-0.20, 0.35)	0.02 (-0.02, 0.05)	0.04 (0.01, 0.08)	0.05 (-0.37, 0.46)	-0.05 (-0.09, -0.00)	-0.00 (-0.03, 0.03)
**Intrauterine devices, high socioeconomic status (lag 0)**							
0.33 (0.21, 0.45)	0.01 (0.00, 0.03)	0.21 (-0.06, 0.47)	0.01 (-0.02, 0.04)	0.03 (-0.00, 0.06)	0.12 (-0.22, 0.46)	-0.05 (-0.08, -0.01)	-0.02 (-0.04, 0.01)
**Oral contraceptives, low socioeconomic status (lag 1)**							
47.53 (40.10, 54.96)	0.01 (-0.74, 0.75)	19.94 (8.70, 31.19)	0.58 (-0.89, 2.05)	0.59 (-0.74, 1.92)	-5.28 (-19.75, 9.19)	-0.99 (-2.87, 0.88)	-0.40 (-1.70, 0.89)
**Oral contraceptives, low-middle socioeconomic status (lag 1)**							
51.98 (45.57, 58.39)	0.25 (-0.36, 0.85)	22.36 (9.20, 35.51)	0.28 (-1.33, 1.90)	0.53 (-1.02, 2.08)	-2.69 (-17.44, 12.07)	-1.19 (-3.08, 0.71)	-0.66 (-1.71, 0.40)
**Oral contraceptives, middle socioeconomic status (lag 0)**							
44.37 (40.02, 48.73)	0.22 (-0.19, 0.62)	18.13 (7.56, 28.69)	0.18 (-1.12, 1.49)	0.40 (-0.84, 1.64)	-0.97 (-13.42, 11.49)	-1.30 (-2.88, 0.29)	-0.90 (-1.88, 0.09)
**Oral contraceptives, middle-high socioeconomic status (lag 2)**							
47.46 (40.56, 54.36)	0.25 (-0.34, 0.85)	17.77 (5.41, 30.13)	0.28 (-1.20, 1.77)	0.54 (-0.89, 1.96)	-1.46 (-14.56, 11.64)	-1.47 (-3.33, 0.39)	-0.93 (-1.97, 0.11)
**Oral contraceptives, high socioeconomic status (lag 2)**							
46.87 (40.23, 53.50)	0.39 (-0.17, 0.95)	15.18 (-1.02, 31.38)	0.16 (-1.69, 2.00)	0.54 (-1.28, 2.37)	-0.43 (-16.42, 15.56)	-1.30 (-3.63, 1.04)	-0.75 (-1.89, 0.39)

© 2025 Downey AK et al. *JAMA Pediatrics*.

eTable 8. Single-group interrupted time series of prescription contraceptives dispensed per 1000 females before and after implementation of OHIP+ and OHIP- in Ontario females 20-24 years-old stratified by SES							
**Pre-policy (Sep 2016 - Dec 2017)**		**OHIP+ (Jan 2018 - Mar 2019)**			**OHIP- (Apr 2019 - Feb 2020)**		
Starting level	Monthly change	Level change	Relative monthly change	Monthly change	Level change	Relative monthly change	Monthly change
Dispensations (95% CI)	Dispensations (95% CI)	Dispensations (95% CI)	Dispensations (95% CI)	Dispensations (95% CI)	Dispensations (95% CI)	Dispensations (95% CI)	Dispensations (95% CI)
**Intrauterine devices, low socioeconomic status lag 0)**							
2.28 (2.10, 2.45)	0.03 (-0.01, 0.06)	1.40 (0.68, 2.11)	0.06 (-0.04, 0.16)	0.09 (-0.01, 0.18)	-1.27 (-2.24, -0.30)	-0.11 (-0.23, 0.00)	-0.03 (-0.09, 0.04)
**Intrauterine devices, low-middle socioeconomic status (lag 1)**							
1.70 (1.50, 1.90)	0.04 (0.01, 0.06)	1.18 (0.72, 1.64)	-0.01 (-0.06, 0.03)	0.03 (-0.01, 0.06)	-0.30 (-0.92, 0.34)	-0.08 (-0.15, -0.01)	-0.05 (-0.12, 0.01)
**Intrauterine devices, middle socioeconomic status (lag 0)**							
1.10 (0.98, 1.21)	0.04 (0.02, 0.06)	0.37 (-0.11, 0.85)	-0.00 (-0.06, 0.05)	0.04 (-0.01, 0.09)	-0.13 (-0.63, 0.36)	-0.08 (-0.14, -0.02)	-0.04 (-0.07, -0.00)
**Intrauterine devices, middle-high socioeconomic status (lag 0)**							
1.02 (0.86, 1.19)	0.05 (0.03, 0.07)	0.68 (0.25, 1.11)	-0.03 (-0.08, 0.02)	0.02 (-0.03, 0.07)	-0.10 (-0.66, 0.47)	-0.02 (-0.10, 0.06)	0.00 (-0.06, 0.07)
**Intrauterine devices, high socioeconomic status (lag 0)**							
0.96 (0.81, 1.12)	0.05 (0.02, 0.07)	0.40 (-0.05, 0.85)	-0.02 (-0.08, 0.04)	0.03 (-0.03, 0.08)	-0.23 (-0.80, 0.35)	-0.03 (-0.08, 0.03)	0.00 (-0.02, 0.02)
**Oral contraceptives, low socioeconomic status (lag 0)**							
98.53 (96.03, 101.02)	-0.72 (-0.99, -0.46)	36.02 (30.18, 41.86)	0.35 (-0.35, 1.06)	-0.37 (-1.03, 0.28)	-3.11 (-12.09, 5.86)	-0.51 (-1.82, 0.79)	-0.88 (-2.01, 0.25)
**Oral contraceptives, low-middle socioeconomic status (lag 0)**							
86.17 (83.94, 88.40)	-0.53 (-0.75, -0.31)	28.49 (23.65, 33.33)	0.07 (-0.51, 0.64)	-0.46 (-1.00, 0.07)	2.29 (-3.35, 7.93)	-0.71 (-1.55, 0.14)	-1.17 (-1.83, -0.51)
**Oral contraceptives, middle socioeconomic status (lag 0)**							
74.25 (71.04, 77.46)	-0.32 (-0.67, 0.03)	20.24 (13.89, 26.59)	-0.04 (-0.75, 0.68)	-0.36 (-0.97, 0.27)	5.32 (-2.27, 12.91)	-0.92 (-1.90, 0.06)	-1.27 (-2.03, -0.52)
**Oral contraceptives, middle-high socioeconomic status (lag 0)**							
78.55 (74.99, 82.11)	-0.29 (-0.70, 0.13)	21.91 (15.18, 28.65)	0.06 (-0.68, 0.81)	-0.22 (-0.84, 0.40)	3.04 (-3.52, 9.61)	-0.94 (-1.76, -0.12)	-1.17 (-1.71, 0.63)
**Oral contraceptives, high socioeconomic status (lag 0)**							
79.18 (74.20, 84.17)	-0.14 (-0.75, 0.47)	20.89 (10.92, 30.86)	-0.26 (-1.33, 0.80)	-0.41 (-1.28, 0.46)	8.83 (-1.48, 19.15)	-1.30 (-2.58, -0.01)	-1.71 (-2.65, -0.76)

© 2025 Downey AK et al. *JAMA Pediatrics*.

eTable 9. Controlled interrupted time series of prescription contraceptives dispensed per 1000 females before and after implementation of OHIP+ and OHIP- for Ontario youth aged 15-19 and 20-24 years-old age-matched Canadian cohorts and stratified by SES					
**Pre-policy (Sep 2016 – Dec 2017)**		**OHIP+ (Jan 2018 – Mar 2019)**		**OHIP- (Apr 2019 – Feb 2020)**	
Difference in starting level	Difference in monthly change	Difference in level change	Difference in monthly change	Difference in level change	Difference in monthly change
Dispensations (95% CI)	Dispensations (95% CI)	Dispensations (95% CI)	Dispensations (95% CI)	Dispensations (95% CI)	Dispensations (95% CI)
**Intrauterine devices – low socioeconomic status 15-19 year-olds, control: Canada[a] 15-19 year-olds (lag 0)**					
-0.72 (-1.13, -0.31)	0.01 (-0.03, 0.06)	-0.01 (-0.41, 0.40)	0.01 (-0.05, 0.06)	-0.05 (-0.54, 0.44)	-0.06 (-0.12, 0.01)
**Intrauterine devices – high socioeconomic status 15-19 year-olds, control: Canada[a] 15-19 year-olds (lag 4)**					
-0.52 (-0.72, -0.32)	0.00 (-0.02, 0.02)	0.17 (-0.20, 0.53)	-0.00 (-0.05, 0.05)	0.14 (-0.24, 0.51)	-0.04 (-0.10, 0.03)
**Intrauterine devices – low socioeconomic status 20-24 year-olds, control: Canada[a] 20-24 year-olds (lag 4)**					
-0.93 (-1.30, 0.57)	0.02 (-0.03, 0.06)	1.21 (0.64, 1.78)	0.03 (-0.05, 0.12)	-1.49 (-2.26, -0.71)	0.01 (-0.07, 0.09)
**Intrauterine devices – high socioeconomic status 20-24 year-olds, control: Canada[a] 20-24 year-olds (lag 0)**					
-0.75 (-1.05, -0.46)	0.04 (0.00, 0.08)	0.31 (-0.27, 0.89)	-0.03 (-0.10, 0.04)	-0.26 (-0.89, 0.38)	0.01 (-0.06, 0.08)
**Oral contraceptives – low socioeconomic status 15-19 year-olds, control: Canada[a] 15-19 year-olds (lag 5)**					
-67.74 (-82.58, -52.90)	-0.29 (-1.71, 1.14)	11.43 (-12.58, 35.45)	0.85 (-1.38, 3.08)	-4.02 (-22.45, 14.42)	-0.49 (-3.70, 2.73)
**Oral contraceptives – high socioeconomic status 15-19 year-olds, control: Canada[a] 15-19 year-olds (lag 5)**					
-57.09 (-68.92, -45.26)	0.10 (-0.96, 1.17)	5.58 (-19.37, 30.52)	-0.10 (-2.40, 2.60)	-0.64 (-19.70, 18.41)	-0.58 (-4.08, 2.92)
**Oral contraceptives – low socioeconomic status 20-24 year-olds, control: Canada[a] 20-24 year-olds (lag 0)**					
-76.84 (-83.04, -70.65)	0.40 (-0.28, 1.08)	14.61 (2.69, 26.53)	0.14 (-1.22, 1.51)	-8.43 (-21.26, 4.40)	-0.30 (-2.19, 1.60)
**Oral contraceptives – high socioeconomic status 20-24 year-olds, control: Canada[a] 20-24 year-olds (lag 0)**					
-48.14 (-55.42, -40.85)	0.43 (-0.42, 1.27)	6.14 (-7.32, 19.60)	-0.07 (-1.56, 1.42)	3.19 (-9.92, 16.30)	-1.08 (-2.79, 0.64)

[a]*Refers to prescriptions dispensed in females from all Canadian provinces, excluding Ontario*

© 2025 Downey AK et al. *JAMA Pediatrics*.

eTable 10. Sensitivity analysis – Single-group interrupted time series of prescription contraceptives dispensed per 1000 females before and after implementation of OHIP+ and OHIP- in Ontario youth under the age of 25, using February 2018 and May 2019 as intervention dates

Pre-policy (Sep 2016 – Nov 2017)		OHIP+ (Feb 2018 – Feb 2019)			OHIP- (May 2019 – Feb 2020)		
Starting level	Monthly change	Level change	Relative monthly change	Monthly change	Level change	Relative monthly change	Monthly change
Dispensations (95% CI)	Dispensations (95% CI)	Dispensations (95% CI)	Dispensations (95% CI)	Dispensations (95% CI)	Dispensations (95% CI)	Dispensations (95% CI)	Dispensations (95% CI)
**Intrauterine devices, Ontario 15-24 years-old (lag 0)**							
0.94 (0.88, 1.01)	0.04 (0.03, 0.05)	0.54 (0.17, 0.91)	-0.01 (-0.06, 0.04)	0.03 (-0.02, 0.07)	0.01 (-0.36, 0.37)	-0.06 (-0.11, -0.00)	-0.03 (-0.06, 0.00)
**Intrauterine devices, Canada[a] 15-24 years-old (lag 0)**							
1.86 (1.72, 2.00)	0.03 (0.01, 0.04)	-0.07 (-0.28, 0.14)	0.00 (-0.03, 0.04)	0.03 (-0.00, 0.06)	-0.00 (-0.31, 0.31)	-0.04 (-0.08, -0.00)	-0.01 (-0.03, 0.01)
**Intrauterine devices, Ontario 25-49 years-old (lag 3)**							
1.44 (1.35, 1.53)	0.02 (0.01, 0.03)	0.11 (-0.04, 0.25)	-0.02 (-0.03, 0.00)	0.01 (-0.01, 0.02)	0.02 (-0.17, 0.20)	-0.01 (-0.04, 0.02)	-0.00 (-0.03, 0.02)
**Oral contraceptives, Ontario 15-24 years-old (lag 0)**							
66.86 (63.41, 70.32)	-0.17 (-0.55, 0.21)	25.15 (17.67, 32.63)	0.03 (-1.10, 1.16)	-0.14 (-1.20, 0.93)	1.01 (-8.68, 10.70)	-1.00 (-2.36, 0.35)	-1.14 (-1.98, -0.31)
**Oral contraceptives, Canada[a] 15-24 years-old (lag 0)**							
143.02 (136.20, 149.85)	-0.51 (-1.12, 0.09)	19.36 (8.17, 30.55)	-0.19 (-1.87, 1.50)	-0.70 (-2.28, 0.87)	5.11 (-7.66, 17.88)	-0.55 (-2.53, 1.44)	-1.25 (-2.46, -0.04)
**Oral contraceptives, Ontario 25-49 years-old (lag 0)**							
36.33 (34.97, 37.68)	-0.14 (-0.29, -0.00)	5.11 (2.95, 7.27)	-0.06 (-0.39, 0.28)	-0.20 (-0.50, 0.11)	0.80 (-1.98, 3.59)	-0.04 (-0.47, 0.39)	-0.24 (-0.54, 0.06)

[a]*Refers to prescriptions dispensed in females from all Canadian provinces, excluding Ontario*

© 2025 Downey AK et al. *JAMA Pediatrics*.

eTable 11. Sensitivity analysis - Controlled interrupted time series of prescription contraceptives dispensed per 1000 females before and after implementation of OHIP+ and OHIP- for Ontario youth under the age of 25 versus control groups, using February 2018 and May 2019 as intervention dates					
**Pre-policy (Sep 2016 – Nov 2017)**		**OHIP+ (Feb 2018 – Feb 2019)**		**OHIP- (May 2019 – Feb 2020)**	
Difference in starting level	Difference in monthly change	Difference in level change	Difference in monthly change	Difference in level change	Difference in monthly change
Dispensations (95% CI)	Dispensations (95% CI)	Dispensations (95% CI)	Dispensations (95% CI)	Dispensations (95% CI)	Dispensations (95% CI)
**Intrauterine devices, control: Canada[a] 15-24 year-olds (lag 0)**					
-0.92 (-1.07, -0.76)	0.01 (-0.01, 0.03)	0.61 (0.18, 1.04)	-0.02 (-0.08, 0.04)	0.01 (-0.47, 0.49)	-0.01 (-0.08, 0.05)
**Intrauterine devices, control: Ontario 25-49 year-olds (lag 0)**					
-0.49 (-0.66, -0.33)	0.02 (0.00, 0.04)	0.43 (0.03, 0.84)	0.00 (-0.05, 0.06)	-0.01 (-0.48, 0.46)	-0.05 (-0.13, 0.03)
**Oral contraceptives, control: Canada[a] 15-24 year-olds (lag 4)**					
-76.16 (-83.45, -68.87)	0.35 (-0.31, 1.00)	5.79 (-7.30, 18.88)	0.22 (-1.48, 1.92)	-4.10 (-15.42, 7.22)	-0.46 (-2.57, 1.66)
**Oral contraceptives, control: Ontario 25-49 year-olds (lag 0)**					
30.54 (26.83, 34.25)	-0.03 (-0.43, 0.38)	20.04 (12.25, 27.83)	0.09 (-1.09, 1.27)	0.21 (-9.88, 10.29)	0.96 (-2.38, 0.46)

[a]*Refers to prescriptions dispensed in females from all Canadian provinces, excluding Ontario*

© 2025 Downey AK et al. *JAMA Pediatrics*.

eTable 12. Sensitivity analysis – Single-group interrupted time series of prescription contraceptives dispensed per 1000 females before and after implementation of OHIP+ and OHIP- in Ontario females 15-24 years-old stratified by SES, using February 2018 and May 2019 as intervention dates

**Pre-policy (Sep 2016 – Nov 2017)**		**OHIP+ (Feb 2018 – Feb 2019)**			**OHIP- (May 2019 – Feb 2020)**		
Starting level	Monthly change	Level change	Relative change	Monthly change	Level change	Relative change	Monthly change
Dispensations (95% CI)	Dispensations (95% CI)	Dispensations (95% CI)	Dispensations (95% CI)	Dispensations (95% CI)	Dispensations (95% CI)	Dispensations (95% CI)	Dispensations (95% CI)
**Intrauterine devices, low socioeconomic status (lag 0)**							
1.47 (1.38, 1.56)	0.04 (0.02, 0.06)	0.86 (0.41, 1.31)	0.01 (-0.04, 0.07)	0.05 (-0.01, 0.10)	-0.42 (-0.82, -0.02)	-0.07 (-0.15, 0.01)	-0.02 (-0.08, 0.03)
**Intrauterine devices, low-middle socioeconomic status (lag 0)**							
1.20 (1.08, 1.33)	0.04 (0.02, 0.06)	0.78 (0.31, 1.24)	-0.01 (-0.07, 0.06)	0.03 (-0.03, 0.09)	0.05 (-0.45, 0.55)	-0.10 (-0.17, -0.03)	-0.07 (-0.11, -0.03)
**Intrauterine devices, middle socioeconomic status (lag 5)**							
0.73 (0.66, 0.80)	0.04 (0.03, 0.05)	0.24 (-0.10, 0.58)	-0.01 (-0.07, 0.04)	0.03 (-0.02, 0.08)	0.12 (-0.25, 0.49)	-0.07 (-0.12, -0.01)	-0.03 (-0.05, -0.02)
**Intrauterine devices, middle-high socioeconomic status (lag 0)**							
0.69 (0.58, 0.79)	0.04 (0.03, 0.06)	0.47 (0.07, 0.87)	-0.03 (-0.08, 0.02)	0.02 (-0.03, 0.07)	0.17 (-0.26, 0.60)	-0.03 (-0.10, 0.04)	-0.01 (-0.06, 0.04)
**Intrauterine devices, high socioeconomic status (lag 0)**							
0.63 (0.56, 0.70)	0.04 (0.02, 0.05)	0.36 (-0.01, 0.73)	-0.03 (-0.08, 0.03)	0.01 (-0.04, 0.06)	0.12 (-0.31, 0.55)	-0.03 (-0.08, 0.03)	-0.01 (-0.04, 0.01)
**Oral contraceptives, low socioeconomic status (lag 0)**							
75.01 (71.56, 78.46)	-0.47 (-0.90, -0.04)	31.39 (24.94, 37.83)	0.33 (-0.67, 1.33)	-0.14 (-1.05, 0.76)	-5.25 (-13.95, 3.45)	-0.25 (-1.61, 1.10)	-0.40 (-1.41, 0.61)
**Oral contraceptives, low-middle socioeconomic status (lag 0)**							
70.38 (67.07, 73.69)	-0.22 (-0.57, 0.13)	28.52 (21.73, 35.31)	-0.01 (-1.06, 1.04)	-0.23 (-1.22, 0.76)	-0.21 (-9.23, 8.81)	-0.71 (-2.00, 0.58)	-0.94 (-1.77, -0.11)
**Oral contraceptives, middle socioeconomic status (lag 0)**							
60.32 (56.83, 6.80)	-0.09 (-0.49, 0.32)	21.52 (13.03, 30.01)	-0.07 (-1.28, 1.14)	-0.16 (-1.30, 0.98)	3.58 (-6.59, 13.75)	-1.31 (-2.68, 0.06)	-1.47 (-2.24, -0.70)
**Oral contraceptives, middle-high socioeconomic status (lag 0)**							
64.24 (60.66, 67.82)	-0.09 (-0.52, 0.34)	22.80 (15.01, 30.59)	0.05 (-1.07, 1.18)	-0.04 (-1.08, 1.00)	0.99 (-8.72, 10.70)	-1.13 (-2.48, 0.22)	-1.17 (-2.02, -0.31)
**Oral contraceptives, high socioeconomic status (lag 0)**							
64.38 (59.91, 68.86)	0.03 (-0.57, 0.62)	21.52 (10.11, 32.92)	-0.14 (-1.65, 1.37)	-0.11 (-1.50, 1.28)	5.93 (-6.59, 18.46)	-1.62 (-3.27, 0.04)	-1.73 (-2.63, -0.83)

© 2025 Downey AK et al. *JAMA Pediatrics*.

eTable 13. Sensitivity analysis - Controlled interrupted time series of prescription contraceptives dispensed per 1000 females before and after implementation of OHIP+ and OHIP- for Ontario youth under the age of 25 versus control groups stratified by SES, using February 2018 and May 2019 as intervention dates					
**Pre-policy (September 2016-November 2017)**		**OHIP+ (February 2018-February 2019)**		**OHIP- (May 2019-February 2020)**	
Difference in starting level	Difference in monthly change	Difference in level change	Difference in monthly change	Difference in level change	Difference in monthly change
Utilization rate (95% CI)	Utilization rate (95% CI)	Utilization rate (95% CI)	Utilization rate (95% CI)	Utilization rate (95% CI)	Utilization rate (95% CI)
**Intrauterine devices – low socioeconomic status, control: Canada[a] 15-24 year-olds (lag 0)**					
-0.82 (-1.02, -0.63)	0.01 (-0.01, 0.04)	0.93 (0.39, 1.45)	-0.01 (-0.09, 0.06)	-0.42 (-1.04, 0.20)	0.01 (-0.08, 0.11)
**Intrauterine devices – low socioeconomic status, control: Ontario 25-49 year-olds lag 0)**					
-0.10 (-0.29, 0.09)	0.01 (-0.01, 0.03)	0.71 (0.23, 1.18)	0.03 (-0.03, 0.10)	-0.36 (-0.87, 0.14)	-0.07 (-0.17, 0.02)
**Intrauterine devices – high socioeconomic status, control: Canada[a] 15-24 year-olds lag 0)**					
-0.64 (-0.80, -0.49)	0.02 (0.00, 0.04)	0.39 (-0.04, 0.82)	-0.04 (-0.10, 0.02)	0.13 (-0.35, 0.61)	0.00 (-0.06, 0.07)
**Intrauterine devices – high socioeconomic status, control: Ontario 25-49 year-olds lag 0)**					
-0.71 (-0.85, -0.57)	0.02 (0.00, 0.04)	0.33 (-0.08, 0.74)	-0.02 (-0.08, 0.05)	0.11 (-0.43, 0.65)	-0.02 (-0.10, 0.06)
**Oral contraceptives – low socioeconomic status, control: Canada[a] 15-24 year-olds (lag 4)**					
-73.44 (-82.58, -64.29)	0.24 (-0.74, 1.22)	12.94 (-0.41, 26.28)	0.27 (-1.14, 1.69)	-7.67 (-17.67, 2.33)	0.02 (-2.17, 2.20)
**Oral contraceptives – low socioeconomic status, control: Ontario 25-49 year-olds lag 0)**					
35.79 (32.00, 39.58)	-0.31 (-0.77, 0.15)	25.21 (18.32, 32.11)	0.38 (-0.69, 1.46)	-6.26 (-15.61, 3.09)	-0.22 (-1.68, 1.23)
**Oral contraceptives – high socioeconomic status, control: Canada[a] 15-24 year-olds lag 0)**					
-52.91 (-60.32, -45.50)	0.39 (-0.42, 1.20)	5.72 (-9.47, 20.91)	-0.04 (-2.16, 2.08)	0.75 (-15.81, 17.30)	-1.03 (-3.40, 1.34)
**Oral contraceptives – high socioeconomic status, control: Ontario 25-49 year-olds lag 0)**					
31.01 (26.34, 35.67)	0.17 (-0.45, 0.78)	17.41 (5.84, 28.98)	-0.09 (-1.63, 1.45)	5.46 (-7.28, 18.19)	-1.61 (-3.30, 0.09)

[a]*Refers to prescriptions dispensed in females from all Canadian provinces, excluding Ontario*

© 2025 Downey AK et al. *JAMA Pediatrics*.
