# Supplementary material for: Universal Pharmacare and Contraceptive Dispensations Among Youth
Source: JAMA Pediatr. 2025 Aug 18;179(10):1090–9. doi: 10.1001/jamapediatrics.2025.2585 (PMC12362276; doi:10.1001/jamapediatrics.2025.2585)
Supplement: Supplement 2. — Data Sharing Statement. [file jamapediatr-e252585-s002.pdf]

Data Sharing Statement

Downey. Universal Pharmacare and Contraceptive Dispensations Among Youth. *JAMA Pediatr*. Published August 18, 2025. doi:10.1001/jamapediatrics.2025.2585

Data
Data available: No
